# Genome-wide association for multiple quantitative traits in forage oat germplasm based on specific length amplified fragment sequencing

**DOI:** 10.3389/fpls.2025.1527635

**Published:** 2025-02-20

**Authors:** Yue Li, Kai Zhu, Huiting Cui, Qiannan Hu, Chu Wang, Fang Jia, Junmei Kang, Chengze Ma, Yan Sun

**Affiliations:** ^1^ Department of Turfgrass Science and Engineering, College of Grassland Science and Technology, China Agricultural University, Beijing, China; ^2^ Institute of Animal Science, Chinese Academy of Agricultural Sciences, Beijing, China; ^3^ School of Agriculture, Henan Institute of Science and Technology, Xinxiang, China; ^4^ Institute of Ecological Conservation and Restoration, Chinese Academy of Forestry, Beijing, China

**Keywords:** *Avena sativa* L., genotyping by sequencing (GBS), node number, flag leaf length, genome wide association analysis (GWAS)

## Abstract

Oats (*Avena sativa* L.) is an ideal forage species due to its excellent production performance, high nutritional value, and robust adaptability. In the present study, we analyzed plant height (PH), panicle length (PL), stem diameter (SD), node number (NN), flag leaf length (FLL), flag leaf width (FLW), second leaf length (SLL), and second leaf width (SLW) in a collection containing 340 forage oat accessions, in order to perform a genome-wide association study (GWAS) for identifying markers associated with the eight traits. We genotyped this collection using specific length amplified fragment sequencing (SLAF-seq). Phenotypes for PH, PL, SD, NN, FLL, FLW, SLL, and SLW were collected under natural conditions in four environments. GWAS analyses detected six significant associations for NN and three for FLL. Candidate genes of the nine associations were screened and discussed. Several genes were found to be associated with node number, including *zinc finger MYM-type protein 1-like isoform X1*, *ervatamin-B-like*, *Pimeloyl-ACP methyl ester carboxylesterase*, and *ACT domain-containing protein ACR4-like*, involved in cell division and organ development. Additionally, three genes were linked to flag leaf length—*putative aquaporin PIP2-2*, *triacylglycerol lipase OBL1-like*, and *scarecrow-like protein 21*—involved in the regulation of plant development and stress response. These SNP markers may be useful to accelerate the breeding progress of forage oat in temperate monsoon environments.

## Introduction

Oats (*Avena sativa* L.), the sixth most important cereal crop worldwide, can be used for both feed and fodder (Reynolds and Suttie, 2004). It is an ideal forage species due to its excellent production performance, high nutritional value, and robust adaptability. In addition, oats serve as a valuable source of human food globally due to their high content of β-glucan, protein, fat, and VB1 (Fu et al., 2020).

In previous studies, based on the current consensus oat map (Chaffin et al., 2016), researchers have paid more attention to grain-related traits, such as lemma color (Wang et al., 2023), seed fatty acid (Carlson et al., 2019) and β-glucan content (Zimmer et al., 2020). Some researchers focused on coleoptile length (Zhou et al., 2024), winter hardness (Wooten et al., 2009), frost tolerance (Tumino et al., 2016), lodging tolerance (Tumino et al., 2017), plant height, and heading date (Tumino et al., 2017; Zimmer et al., 2018; Castilla et al., 2021).

The research on traits for forage use, such as leaf length, node number, panicle length, and stem diameter, has been ignored for a long time, and only a few studies were reported. For a forage grass, these vegetative traits are directly or indirectly related to the formation of forage yield and quality. For example, plant height and the number of nodes on the main stem as key plant-type traits have obvious effects on yield because they are related to some important characteristics such as lodging and adaptability (Chapman et al., 2003; Liu et al., 2011). After that, the size of the flag leaf comprising length, width, and area is an important factor determining plant architecture and potential yield (Duncan, 1971; Guitman et al., 1991; Sharma et al., 2003). Thus, flag leaf is regarded as the “functional leaf” and contributes 45%–58% of plant photosynthate and 41%–43% of the carbohydrates during the grain-filling period of wheat (Sourdille et al., 2002; Sharma et al., 2003; Khaliq et al., 2008; Pérez-Pérez et al., 2010). Leaf quantity is directly related to crude protein content which is one of the important indicators for evaluating the nutritional quality of forage. Node number could affect plant height (Heuer et al., 2001), which is correlated with grass yield positively (Huang et al., 2020). So, more attention should be paid to vegetative traits, especially in forage crops.

In recent years, plant breeding has experienced significant advancements due to the integration of molecular biology techniques. One such technique is marker-assisted selection (MAS), which has become a promising tool for plant breeders worldwide. MAS uses genetic markers associated with specific traits to improve the efficiency and precision of the plant selection process in breeding programs. However, in the field of forage oat breeding, the MAS-based breeding strategy is still immature because of its huge and complicated genome. The cultivated oat is hexaploid, based on a base number of seven chromosomes. Recently, the reference genome of naked oats was published to be 10.8 Gb (Peng et al., 2021), and the hulled oat genome was released on the website of the National Center for Biotechnology Information (National Center for Biotechnology Information (NCBI) Avena sativa genome assembly Oat_OT3098_v2, 2024). The precious genomic information will accelerate the MAS-based breeding of oats.

Traditional genotyping methods for oat germplasm screening typically involved techniques such as PCR-based markers, including RAPD, RFLP, and SSR. These methods were useful but had limitations in terms of throughput, cost, and the ability to capture the full range of genetic variation. Genotyping-by-sequencing (GBS) and similar methods based on DNA sequencing technology have revolutionized the development of molecular breeding. Specific length amplified fragment sequencing (SLAF-seq) is an economic and high-throughput genotyping method similar to GBS, which is based on next-generation sequencing (NGS) (Zhou and Pan, 2023). NGS-based methods, such as GBS and SLAF-seq, offer several advantages over traditional methods, such as high throughput, high resolution, and flexibility. It has been applied in many species of plants, such as sugarcane (*Saccharum* spp.) (Zhang et al., 2022), *Miscanthus* (Chen et al., 2022), and wheat (Wang et al., 2022), and single nucleotide polymorphism (SNP) markers based on SLAF have been utilized in genome-wide association studies (GWAS) of *Astragalus adsurgens* (Gong et al., 2022).

NGS-based methods allow for the simultaneous sequencing of large numbers of samples, greatly increasing the efficiency and speed of genotyping. NGS-based methods provide a more detailed and comprehensive view of the genome, allowing for the identification of a larger number of genetic markers and variants. NGS technologies can be customized to target specific regions of the genome, providing more flexibility in genotyping strategies. In the context of cost-effectiveness, despite the large size of the oat genome and currently the high costs of sequencing and data analysis, the advantages of NGS-based methods will become increasingly evident as sequencing costs continue to decrease.

Given the importance of vegetative traits for forage oat yield and quality and the insufficient studies in genetics of these traits, this study aims to identify SNP markers and genes associated with vegetative traits through a GWAS conducted on a panel of 340 diverse genotypes.

## Materials and methods

### Experimental materials and site description

The 340 forage oat germplasm resources utilized in this experiment were preserved by the lab of Sun Yan from the College of Grassland Science and Technology at China Agricultural University (Beijing). Among these germplasm materials, 282 were sourced from the National Crop Germplasm Resource Center of China (Beijing), 28 from the Qinghai Academy of Animal Husbandry and Veterinary Sciences (Qinghai Province), and 15 from the Seed Resource Medium-term Repository of the Chinese Academy of Agricultural Sciences (Inner Mongolia Autonomous Region); 10 were purchased from Beijing Zhengdao Ecological Technology Co., Ltd.; 3 were purchased from Beijing Crowgrass Technology Development Center; and 2 were purchased from BaiLv Group (Jiangsu Province). All germplasm materials were planted in 2022 and 2023, respectively, in the Changping Experimental Base in Beijing (116.23°E, 40.17°N) and the Pingluo Experimental Base in Ningxia Hui Autonomous Region (106.59°E, 38.95°N).

### Experimental design

The experiment adopted a drilling planting method with a row spacing of 30 cm, and the sowing density was 6,400,000 seeds per hectare. Irrigation was applied once after sowing, and urea was top-dressed at the jointing stage at a rate of 30 g/m². Phenotypic data such as plant height (from the surface of the soil to the tip of the plants), panicle length (from the base to the top of the panicle), stem diameter (in the middle of stem between the first and second node near the root), node number (the total number of nodes from the root to the top of the plant), length (from the bottom to the tip of the leaf), and width (in the direction perpendicular to the middle of the leaf area, the widest part of the leaf was measured) of the flag leaf and the second-to-last leaf were measured in this study. These eight phenotypic traits were selected because they can basically depict the overall phenotypic characteristics of oat germplasm. Plant height reflects the maximum height that oat plants can reach by the milk-ripe stage, panicle length approximately indicates the size of the plant’s panicle, leaf length and width describe the morphological characteristics of the plant’s leaves, and stem diameter and number of stem nodes reflect the characteristics of the plant’s stem.

When the forage oats reached the milk-ripe stage, yield-related agricultural traits were measured. Five plants from the middle of each row were selected to measure plant height, panicle length, stem diameter, node number, and the length and width of the flag leaf and second-to-last leaf.

### Phenotype measurement and data analysis

Years of multisite phenotype data were converted into best linear unbiased prediction (BLUP) data, with BLUP calculations performed using in-house R scripts. The mathematical model for the BLUP transformation is as follows:


$$Y_{jkh}=m+l_{k}+g_{j}+y_{h}+gl_{jk}+gy_{jh}+e_{jkh}$$

Where $Y_{jkh}$ is the observation of individual *i*th in the *h*th year at the *k*th location, m is the mean value, $l_{k}$ is the effector at the *k*th location, $g_{j}$ is the effector of the *j*th genotype, $\;y_{h}$ is the effector in the *h*th year, $gl_{jk}$ is the interact effector between genotype and location, and $gy_{jh}$ is the interact effector between genotype and year. $e_{jkh}$ is residuals. The line BLUPs will be used for subsequent GWAS.

### DNA extraction and WGS sequencing

A representative individual was selected from the field, and leaf samples were taken back to the laboratory for genomic DNA extraction using the TIANGen DNA Extraction Kit (Yuntai Biotechnology Co., Ltd, Beijing). After extraction, the quality and concentration of the DNA were assessed using a NanoDrop 2000 ultraviolet-visible spectrophotometer (Thermo Fisher Scientific, USA). Once the DNA samples passed quality control, the SLAF genome sequencing process was carried out. First, the qualified genomic DNA samples were digested with the restriction enzyme *Hae*III. The resulting enzyme-digested fragments (SLAF tags) were treated with 3′-end A addition, ligation of dual-index sequencing adapters, PCR amplification, purification, pooling, and size selection of DNA fragments with lengths of 414–444 bp by gel electrophoresis, which were defined as SLAF tags. After library quality inspection, sequencing was performed with the help of the NovaSeq 6000 sequencing platform at Beijing Biomarker Technologies Corporation. Each sample had approximately 10 Gb of sequencing data, with a Q30 sequencing quality of 85%. The raw sequence data reported in this paper have been deposited in the Genome Sequence Archive (Chen et al., 2021) in the National Genomics Data Center (Memberspartners, 2021), China National Center for Bioinformation/Beijing Institute of Genomics, Chinese Academy of Sciences (GSA: CRA019491) that are publicly accessible at https://ngdc.cncb.ac.cn/gsa.

### SNP marker detection

The sequencing data were filtered with Trimmomatic software (Bolger et al., 2014), and sequencing adapters, primer sequences, and low-quality reads were removed. Subsequently, the BWA-MEM (v0.7.10-r789) software (Li, 2013) was employed to align the filtered data to the reference genome. The SAMtools (v1.9) software (Li et al., 2009) was then employed to convert SAM files generated from a previous alignment into sorted BAM files. The Picard (v1.94) software (Toolkit, 2019) was employed to mark duplicate sequences, and the GATK (v3.8) software (Van der Auwera et al., 2013) was utilized to correct indel interference with SNP results. SNPs were detected by the SAMtools (v1.9) (Li et al., 2009) software. The default parameters were used for all software mentioned above. Finally, VCFtools (v0.1.15) (Danecek et al., 2011) was employed for filtering, with criteria of less than 10% missing data, a minor allele frequency (MAF) of at least 0.05, and a minimum sequencing depth of 5.

### Population structure analyses

The population structure analysis of forage oats was performed using the Admixture software, with the optimal number of subpopulations (*K*) determined using cross-validation (CV error) assessment. The clustering type with the smallest CV error was identified as the optimal population structure. The population structure visualization was created using the R package pophelper (Francis, 2017). Principal component analysis (PCA) was performed using GAPIT3, and the PCA results were visualized using the R package ggplot2 (Wickham, 2011).

### Genome-wide association study

GWAS was performed using the BLINK model in the GAPIT3 software (Huang et al., 2019; Wang and Zhang, 2021), with the first 20 principal components (PC1–20) included as covariates to control for population structure. We also used other models to perform the association analysis, such as the GLM, MLM, SUPER, CMLM, and MLMM models, but the Q-Q plot showed that none of these models performed better than the BLINK model in this study (**Supplementary Figures S1**, **S2**). A Bonferroni correction was applied to set the significance threshold for the association analysis at 8.30^−8^ (0.05/602572). The results were visualized using Manhattan and Q-Q plots generated by the GAPIT3 software.

### Selection of candidate genes

Firstly, the TBtools software (Chen et al., 2023) was used to acquire the positional information of all genes within a 200-kb range upstream and downstream of the SNP loci from the genome annotation file. Subsequently, based on this positional information, the genome sequences of all target genes were retrieved from the publicly available oats genome sequence on the NCBI website. Finally, the BLASTP was employed to obtain annotation information for these sequences.

### Development of a core collection

A core collection was sampled from the whole collection using the sampleCore function from the Core Hunter 3 package (Thachuk et al., 2009). The genotypic and phenotypic data, including the traits SLL, FLL, SLW, FLW, PH, PL, SD, and NN, were used as input data together. The size of the core collection was set as 10% and 15% of the whole collection, respectively. Moreover, we employed the evaluateCore function in the Core Hunter 3 package to evaluate the proportion of alleles observed in the full genotypic dataset that is retained in the selected core collection. Furthermore, a PCA analysis was conducted for our collection to illustrate the distribution of the chosen samples within the proposed core collection.

## Results

### Evaluation of phenotypic traits

**Figure 1:**
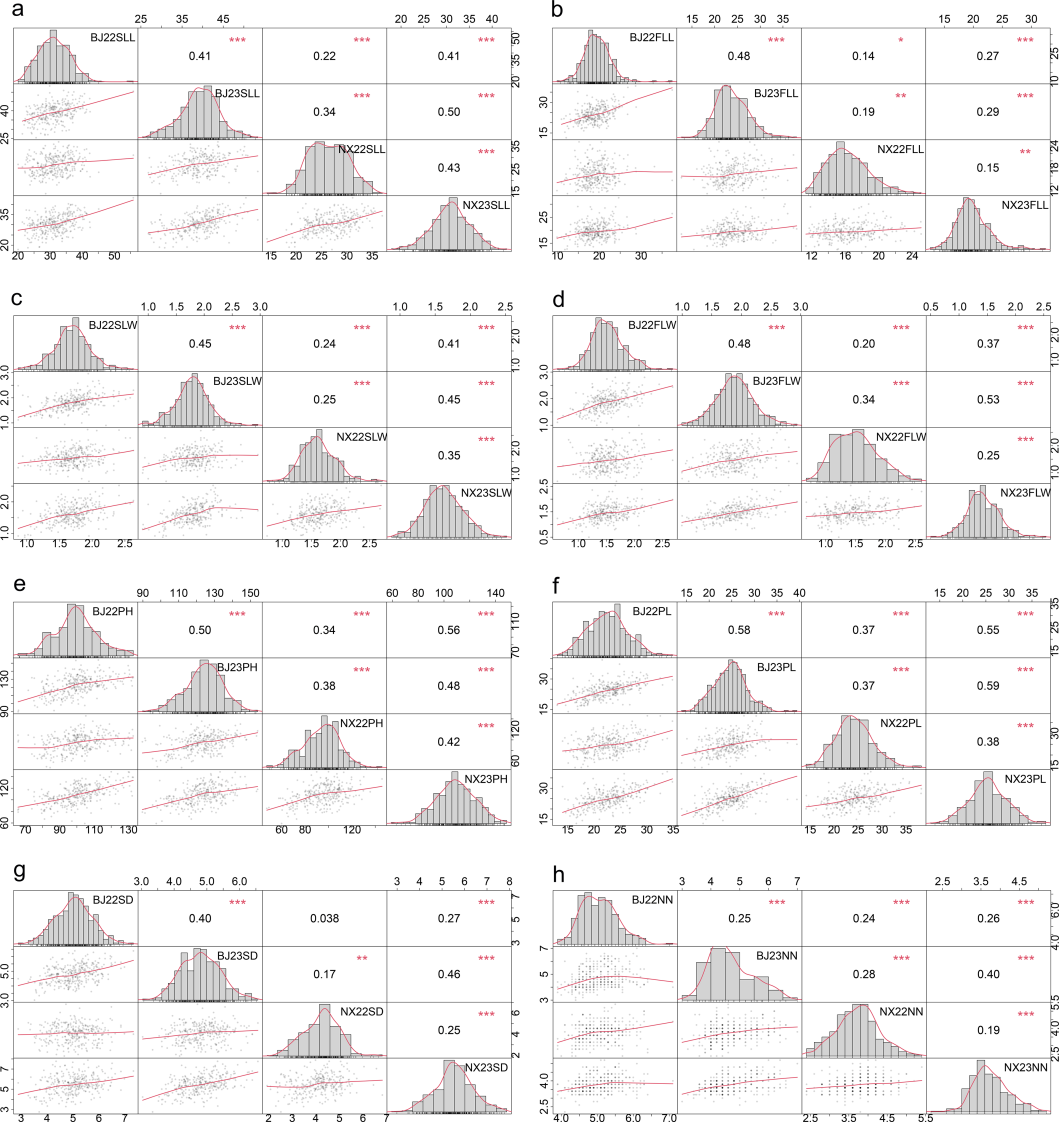
Frequency distribution and correlation of SLL **(A)**, FLL **(B)**, SLW **(C)**, FLW **(D)**, PH **(E)**, PL **(F)**, SD **(G)**, and NN **(H)** between different environments. * means significance <0.01, ** means significance <0.001, *** means significance <0.0001. BJ, Beijing; NX, Ningxia; 22, 2022; 23, 2023; SLL, second-to-last leaf length; SLW, second-to-last leaf width; FLL, flag leaf length; FLW, flag leaf width; PH, plant height; PL, panicle length; SD, stem diameter; NN, node number.

A total of 340 accessions were evaluated under field conditions, and the eight traits were screened for assessment. The distributions of the eight traits were approximately normal in all assessed environments (**Figure 1**). The correlation analysis of the eight traits of test materials in different years and different locations was carried out. As shown in **Figure 1**, the phenotypic correlations of seven traits of the test germplasm materials except stem diameter were significantly correlated in different years and different locations (*P* < 0.05). There was no significant correlation between the stem diameter in Beijing in 2022 and that in Ningxia in 2022.

In order to detect the phenotypic differences of individuals of the same germplasm material under different environmental conditions, ANOVA was performed on the phenotypic data obtained under four environments. As shown in **Supplementary Table 1**, there were extremely significant differences in phenotype between different environmental conditions (*P <* 0.0001). Tests of genotype × location and genotype × year interaction on original traits showed significant genotype × location and genotype × year interaction (**Supplementary Table 1**), respectively, for the eight traits. Although there was a significant genotype × year interaction and a significant genotype × location interaction, data were analyzed jointly because most of the observations were significantly correlated across four environments (**Figure 1**), and this enables the identification of stable alleles across environments.

### Statistical analysis of SNP data

**Figure 2:**
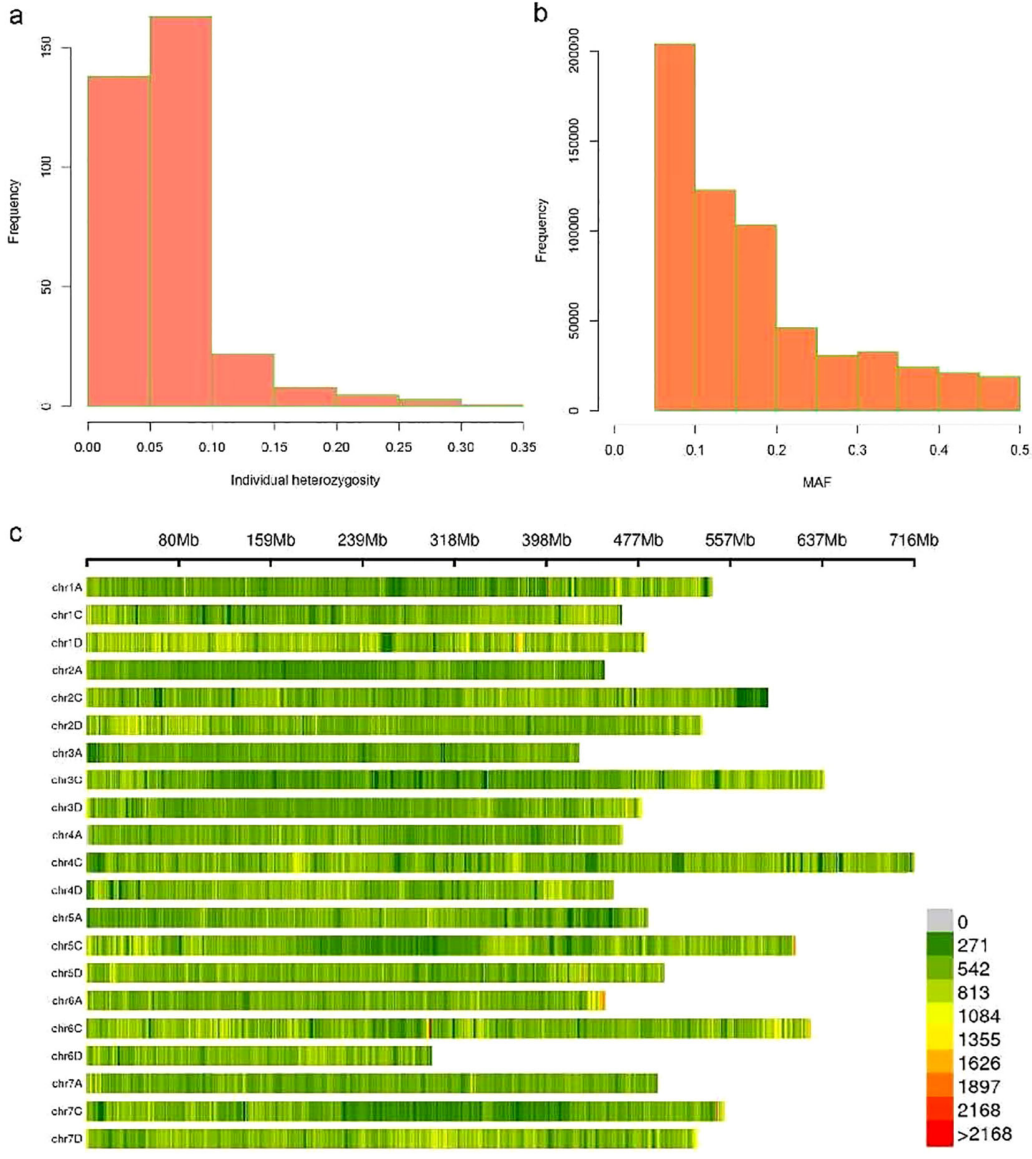
The basic statistics information of SNP. **(A)** Individual heterozygosity is displayed by histogram. **(B)** MAF distribution is shown in a histogram. **(C)** Marker distributions are displayed as a heatmap on 21 chromosomes.

A total of 5,918,177 population SNP markers were identified in this study. SNPs were filtered using the criteria of minor allele frequency (MAF) ≥0.05, missing rate <10, and minimum sequencing depth >20. There were 602,572 high-quality markers obtained. The individual heterozygosity of most samples is less than 0.1 (**Figure 2A**). The distribution of minor allele frequency showed a decrease in the number of markers as the MAF value increased, with the highest number of markers falling within the MAF range of 0.05~0.1 (**Figure 2B**). Marker distributions are displayed as a heatmap on 21 chromosomes (**Figure 2C**). The average SNP density was 17,652.86 bp/SNP.

### Analysis of population structure

**Figure 3:**
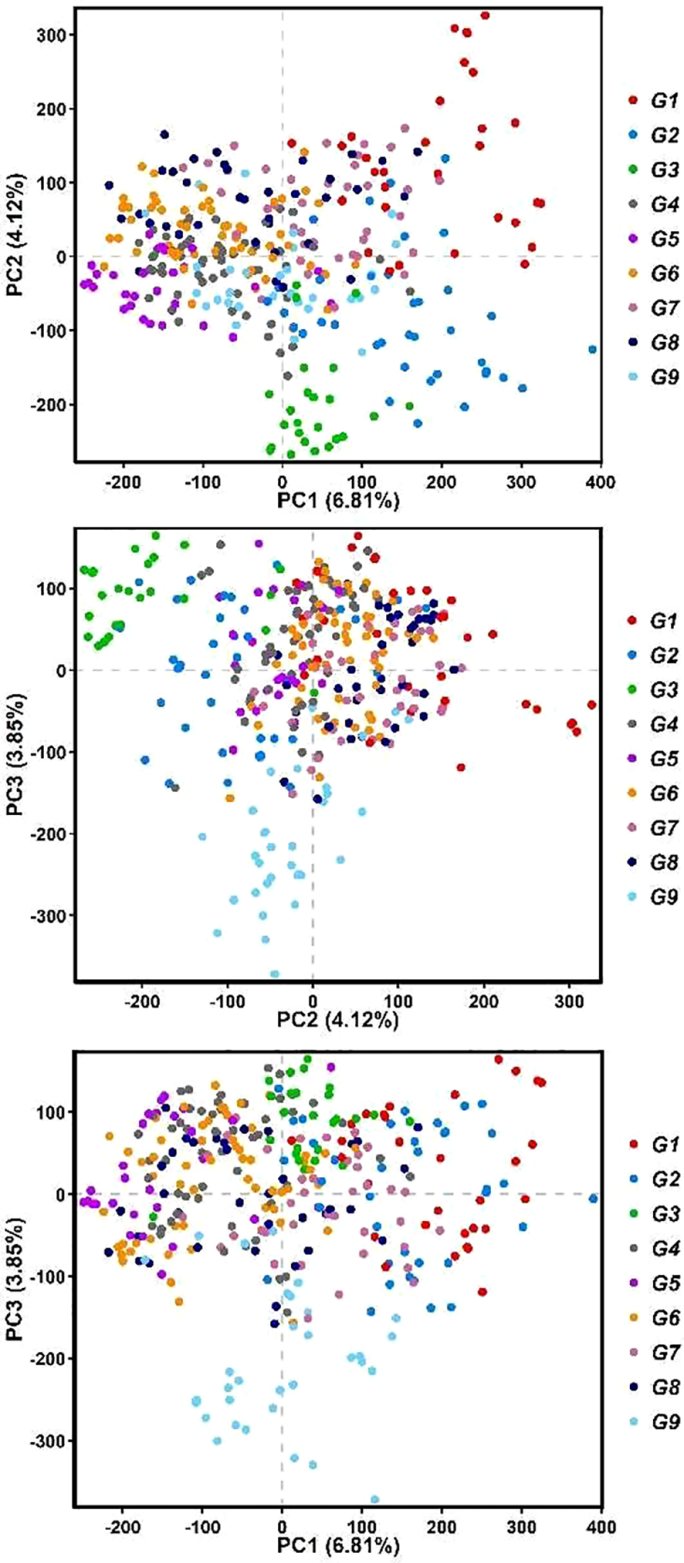
The principal analysis of forage oat accessions. Red dots represent group 1 (G1), blue dots represent group 2 (G2), green dots represent group 3 (G3), gray dots represent group 4 (G4), purple dots represent group 5 (G5), orange red dots represent group 6 (G6), pinkish purple dots represent group 7 (G7), black dots represent group 8 (G8), and sky blue dots represent group 9 (G9).

A total of 602,572 high-quality SNPs were used for the population structure analysis, and we conducted PCA to assess the genetic diversity of forage oat germplasm resources. The results revealed that the first three principal components, PC1, PC2, and PC3, explained 6.81%, 4.12%, and 3.85% of the genetic variation, respectively, and a total of 14.78% of the genetic variation (**Figure 3**). The principal component analysis revealed minimal variation among the germplasm samples, implying a subtle population structure. This finding mirrors previous research on globally diverse oat germplasm, where similarly weak population structures were observed (Newell et al., 2011).

**Figure 4:**
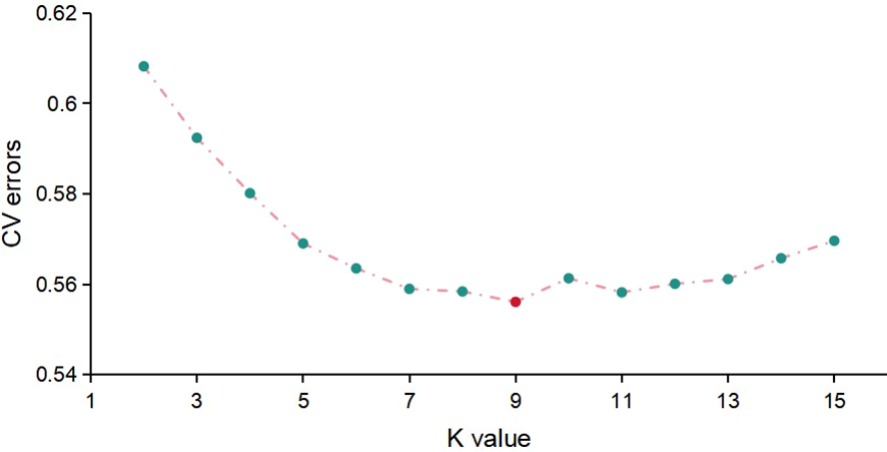
The CV error of different *K* values of population structures.

**Figure 5:**
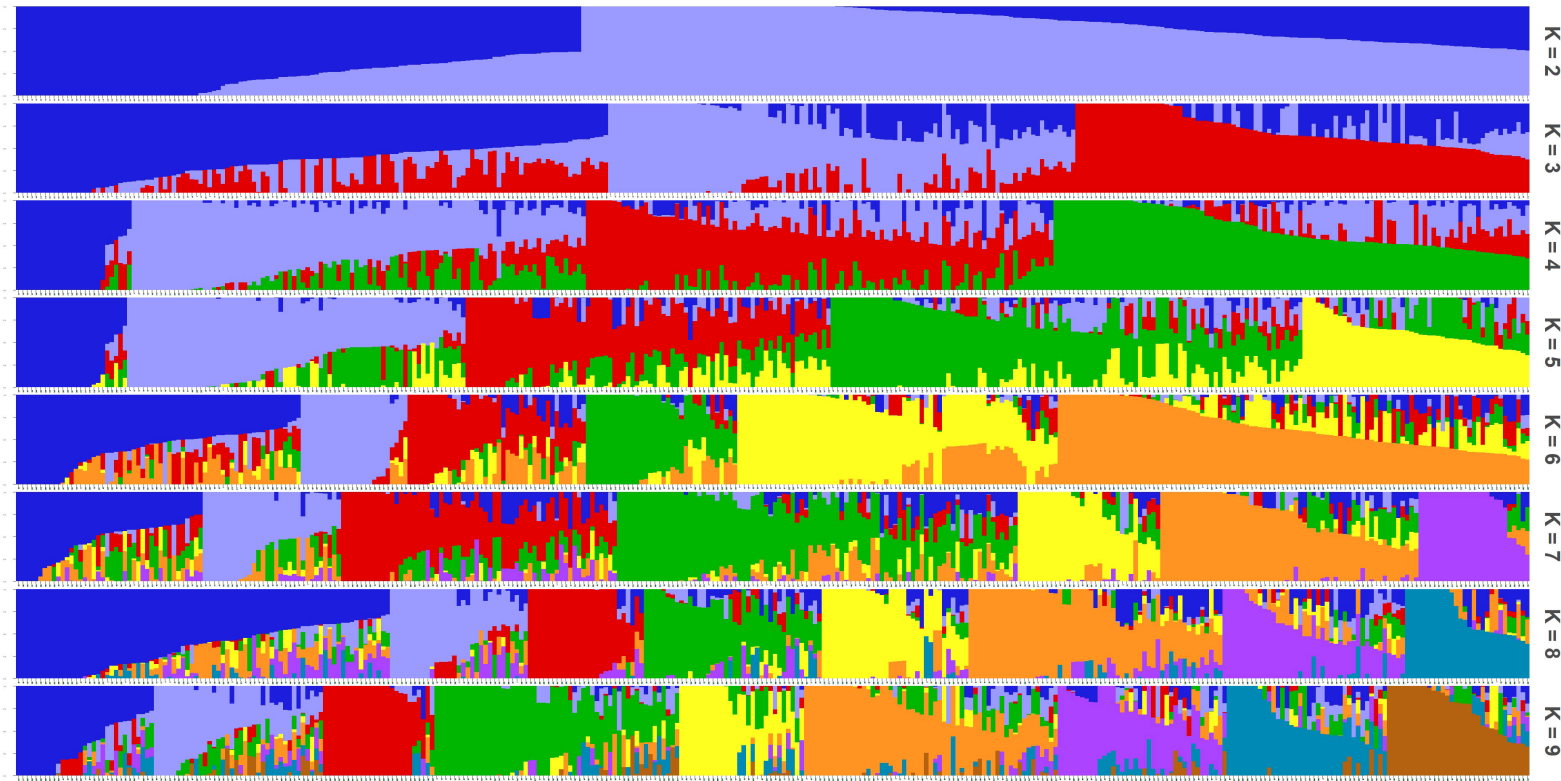
The admixture of population structures. The subgroup is illustrated for *K* = 2 to *K* = 9 for accessions. Accessions are sorted based on the admixture with *K* = 2.

The Admixture software was employed to assess population structure by calculating the CV error across a range of subpopulation numbers (*K*) from 2 to 10. The results indicated that the minimum CV error occurred at *K* = 9, suggesting an optimal subpopulation composition (**Figure 4**). The population structure diagram, which showed changes from two to nine subpopulations, illustrated increasing mixture between populations with higher *K* values (**Figure 5**). According to the clustering results, apart from N22, N23, N27, N31, N41, and AN254, more than 20 naked oat materials were assigned to the same group.

### Genome-wide association analysis of node number and flag leaf length

**Figure 6:**
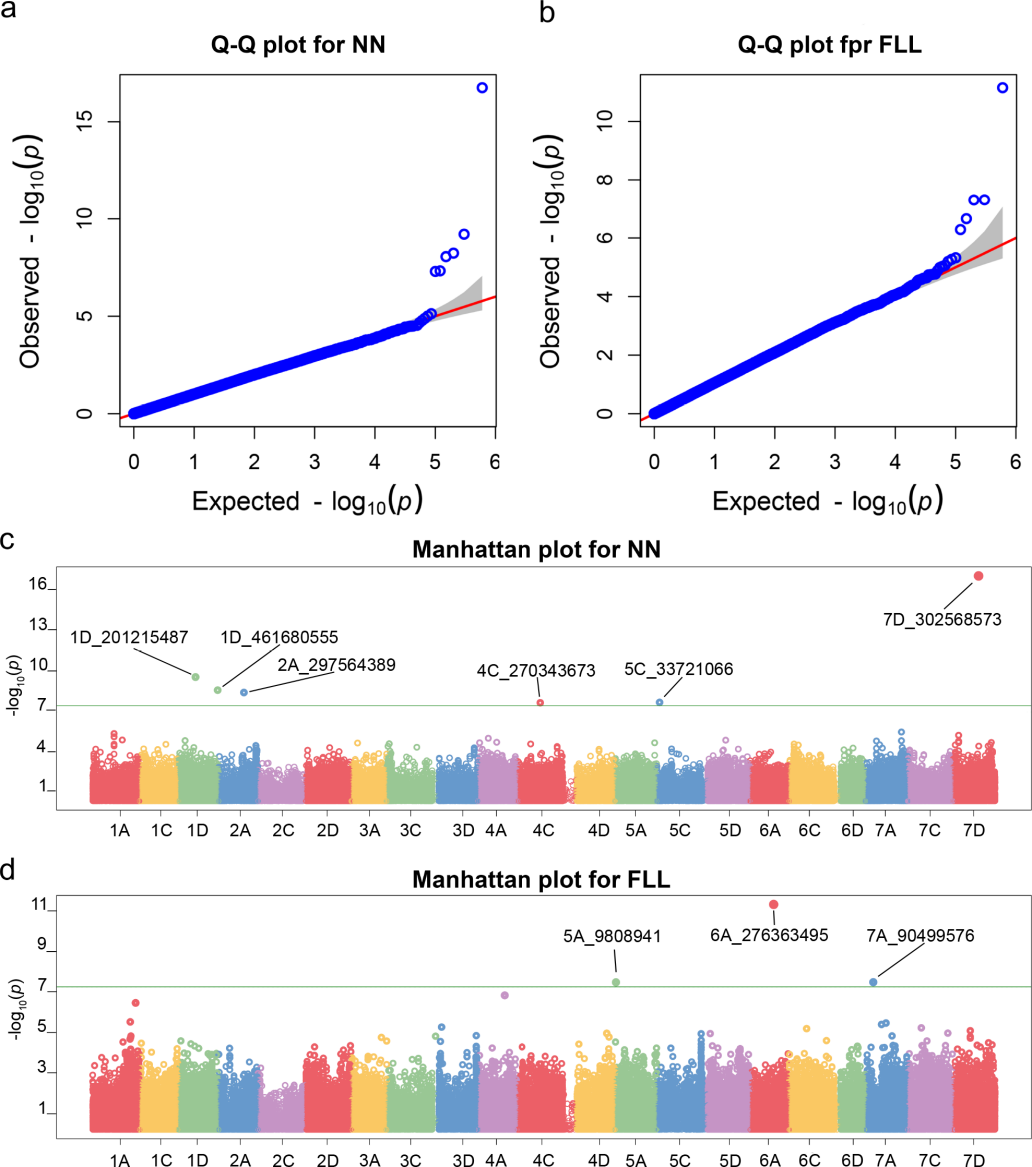
Q-Q plot for FLL **(A)** and NN **(B)** of BLUPs and Manhattan plot for FLL **(C)** and NN **(D)** of BLUPs.

In this study, phenotypic data of forage oats were collected from four different environments: Changping (Beijing) in 2022 and 2023 and Pingluo (Ningxia Hui Autonomous Region) in 2022 and 2023. These phenotypic values were then converted into BLUP values, which were used for the GWAS analysis. The results of the GWAS analysis are shown in **Figure 6**, revealing a total of six SNP loci significantly associated with node number (NN) (**Figure 6C**) and three SNP loci significantly associated with flag leaf length (**Figure 6D**). Two SNP loci (1D_201215487 and 1D_461680555) were located on chromosome 1D, one SNP locus (2A_297564389) on chromosome 2A, one SNP locus (4C_270343673) on chromosome 4C, one SNP locus (5C_33721066) on chromosome 5C, and one SNP locus (7D_302568573) on chromosome 7D. Among these, the locus 7D_302568573 exhibited the lowest *P*-value and the highest correlation with NN (**Table 1**).

**Table 1:** Significant SNP markers and candidate genes associated with NN and FLL.

Traits	Marker	CHR	SNP position	Gene ID	Gene/maker distance (kb)	Position relative to the marker	Gene length	Strand	*P*-value	Description	Function	Species	Cov%	E-value	Ident%	Accession number
NN	7D_302568573	7D	302,568,573	*7D_gene23644*	90.1	Upstream	697	+	1.79E−17	Hypothetical protein QYE76_059829	Unknown	*Lolium multiflorum*	91%	2E−106	70.56	KAK1642024.1
NN	1D_201215487	1D	201,215,487	*1D_gene11508*	28.2	Downstream	1,077	−	6.23E−10	Zinc finger MYM-type protein 1-like isoform X1	Transcript factor	*Triticum dicoccoides*	94%	1E−152	66.67	XP_037407775.1
NN	1D_461680555	1D	461,680,555	*1D_gene55679*	14.3	Upstream	1,253	+	5.85E−09	Ervatamin-B-like	Cysteine protease activity	*Lolium rigidum*	86%	0	73.96	XP_047076862.1
NN	2A_297564389	2A	297,564,389	*2A_gene19938*	10.7	Upstream	3,709	+	8.78E−09	Pimeloyl-ACP methyl ester carboxylesterase	Biotin biosynthesis	*Lolium rigidum*	30%	2.00E−156	69.5	XP_047065907.1
NN	5C_33721066	5C	33,721,066	*5C_gene3312*	21.9	Upstream	2,335	+	4.72E−08	ACT domain-containing protein ACR4-like	Flower and root development; cell division	*Lolium rigidum*	68%	1.00E−81	88.1	XP_047092607.1
NN	4C_270343673	4C	270,343,673	*4C_gene32033*	9.7	Downstream	521	−	5.04E−08	hypothetical protein EJB05_32782	Unknown	*Eragrostis curvula*	36%	1.00E−28	85.94	TVU23052.1
FLL	6A_276363495	6A	276,363,495	*6A_gene22964*	8.1	Upstream	5,582	−	7.07E−12	putative aquaporin PIP2-2	Associated with abiotic stress; water absorption; fiber elongation; photosynthesis	*Triticum urartu*	23%	1.00E−111	81.78	EMS56715.1
FLL	7A_90499576	7A	90,499,576	*7A_gene17036*	52.5	Downstream	860	+	4.87E−08	Triacylglycerol lipase OBL1-like	Metabolism of fatty acids and glycerol	*Lolium perenne*	99%	2.00E−166	81.12	XP_051204323.1
FLL	5A_9808941	5A	9,808,941	*5A_gene1085*	102.7	Downstream	1,616	+	4.93E−08	Scarecrow-like protein 21	Shoot and root growth	*Lolium rigidum*	99%	0	89.76	XP_047089721.1

Three SNP loci significantly associated with flag leaf length were located on chromosomes 5A (5A_9808941), 6A (6A_276363495), and 7A (7A_90499576) (**Figure 6D**). Among these, locus 6A_276363495 exhibited the lowest *P*-value and the highest correlation with flag leaf length (**Table 1**). Additionally, GWAS analysis was performed on six other phenotypes: plant height, spike length, second-to-last leaf length and width, flag leaf width, and stem diameter. However, no significant SNP loci associated with these traits were identified. We screened SNP loci whose *P <*10^−5^, and then we got 8, 18, 9, 10, 9 and 8 SNPs associated with FLW, PH, PL, SD, SLL, and SLW, respectively. The detailed location of the SNP in chromosomes and the *P-*value are provided in **Supplementary Table S3**.

**Figure 7:**
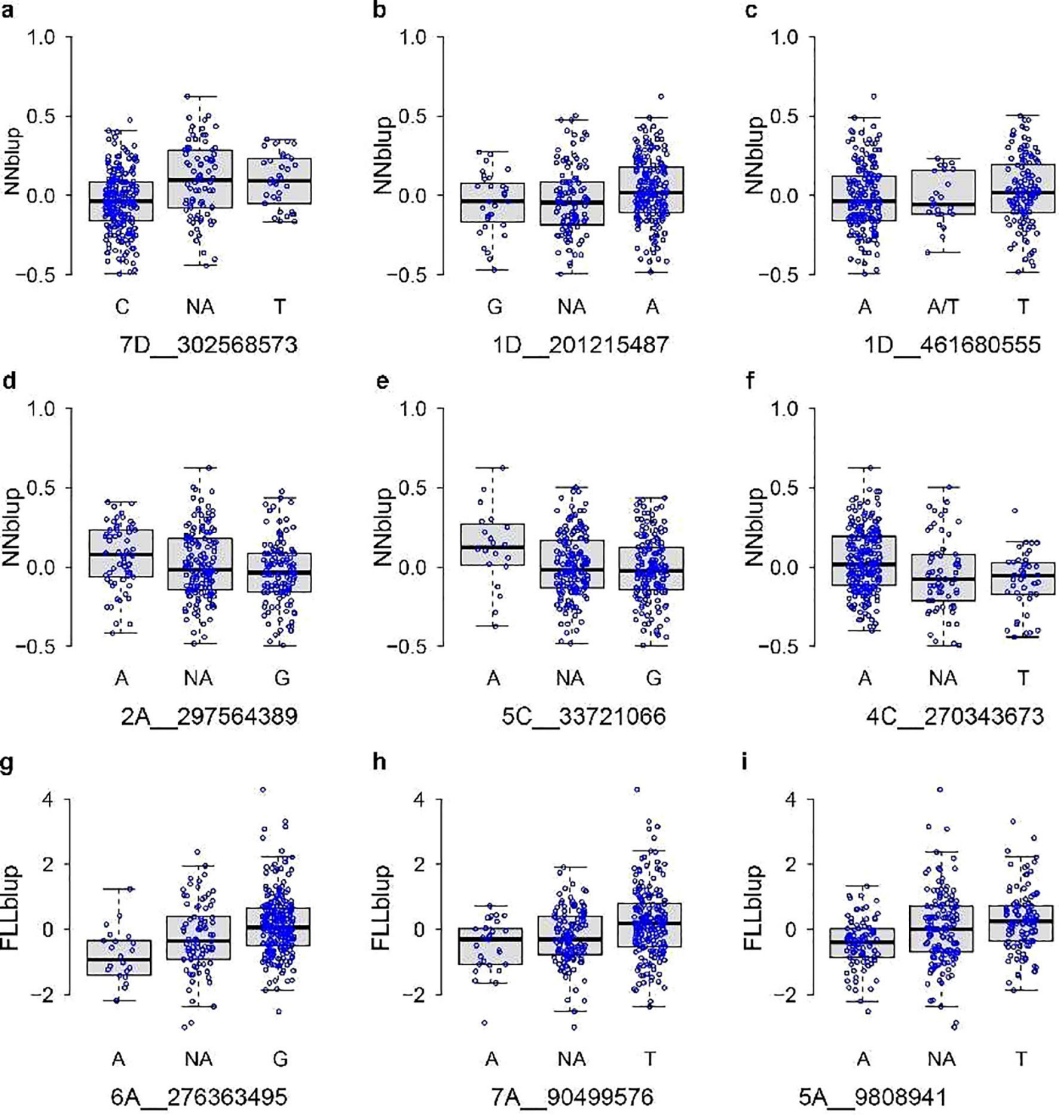
Boxes of FLL and NN distribution for different genotypes associated with the SNP loci. NN distribution for different genotypes associated with 7D_302568573 **(A)**, 1D_201215487 **(B)**, 1D_461680555 **(C)**, 2A_297564389 **(D)**, 5C_33721066 **(E)**, and 4C_270343673 **(F)**. FLL distribution for different genotypes associated with 6A_276363495 **(G)**, 7A_90499576 **(H)**, and 5A_9808941 **(I)**. The *x*-axis and *y*-axis labels denote the SNP marker and BLUP of traits, respectively. NA means that the heterozygous genotype was missing in the test population.

Phenotypic differences among various genotypes were indicated in boxplots (**Figure 7**). Notably, several SNP markers, such as eight out of the nine SNP loci associated with stem node number and flag leaf length (1D_201215487, 2A_297564389, 4C_270343673, 5C_33721066, 7D_302568573, 5A_9808941, 6A_276363495, 7A_9049576), exhibited a lack of heterozygous genotypes in the test population. These observations indicated a relatively high level of homozygosity within the tested population. The sequences corresponding to the 50-bp regions upstream and downstream of the SNP markers are provided in **Table 2**.

**Table 2:** Flanking sequence information of traits associated with SNP.

Traits	SNP	Variants	Flanking sequence
NN	7D_302568573	C/T	AACCCTTCCAGTGGTGAAGTCGGTACGGGTGTGTGGTATGGTCACGCGCG **C** CCATATGACTGGTCATTACATGTCCAGTATATTTTCTTTAATTCTAATGT
NN	2A_297564389	G/A	CTAGCAATGTGGGACTAAACATTTGTCCCAACTCTTGCCTTGCATGAATG **G** GCCACATGGGCCTCTAGGATTTTCAGGAATTATTGAAATAGAAAATGGGC
NN	1D_461680555	A/T	AGTAAGGAAATATTCTCGGTATTGGACGGGACAAAAGCCAAAGTCAATAA **C** TTTCCGTGGGGAACATGAAGTCCAGAGGGGAGACGGAGAGGCGTGCCAAG
NN	4C_270343673	A/T	AGGCCATCCATTCCCTCCTTCATCCTCACACCTCCTTGTGAAGGATCTCT **C** TCCCCTCTAAAATTATTCCCCCATGGTCTCGGATTAGCGAGATACATGTT
NN	1D_201215487	G/A	TCGGAATTGGACGGAACAAAAGCCAAAGTCAATATTTTACCGTGGGAGTC **G** TGAAGCTAGAAGGGGAGACAAAGAGCTGCCAGAGGGCCACCACACCCCCA
NN	5C_33721066	G/A	AGCTCGAGTACCTGTAGGGTATACCCATGTCAGTAGCCCCCGAGTGTCCA **G** TGGAATCACAGATTTGACTAGGGACCTTTCTGAAGTCTTATGAGTGCATG
FLL	5A_9808941	T/A	GCCCTAGAACCATAAAATTGTGGGCAATAGCTCATGAAAACAGCCATAAA **T** CGGGAAAACTACGAGTTTTTTTGTCCTATATATCAAACATGTAGCGGGTC
FLL	6A_276363495	G/A	CTCGTCCGGTATGACACACTTACTCGTAGCCGTGGACAAGTTCACCAAAT **G** GATCGAGGCGAAGCCAATCAAGAAGCTGGACGGAGCGACGGTGATCAAGT
FLL	7A_90499576	T/A	AGATTGAAGCGATACTTTTGGCGCTCGAAATTGCTGCAGCCGTCTGTACC **A** ATGTGCATGCACTAAGCTGATTTTGGTGGGGCCCACTGTATATACACGTA

The variance location of SNP in sequences was marked by an underline. Sequence information covers left and right 50 bp of the SNP. The variance loci of the sequence may be different with the reference allele. The reference allele and the alter allele are unphased genotype.

### Analysis of candidate genes controlling NN and FLL in oats

Based on previous studies on linkage disequilibrium (LD) in forage oats, the LD distance in the forage oat genome is approximately 200 kb (Newell et al., 2011). Thus, we extracted 200 kb interval information upstream and downstream of the associated SNP loci, and then candidate genes were identified. Within the above interval, several genes linked to six SNP loci associated with stem node number were identified, and the annotation information of the gene closest to the associated SNP locus is shown in **Table 1**.

The marker 7D_302568573, associated with NN, was localized to the 7D chromosome at position 302568573 bp. The closest gene named *7D_gene23644* was detected at 90.1 kb upstream of the associated marker. This gene showed similarity with the hypothetical protein QYE76_059829 of *Lolium multiflorum* which was validated by BLASTP.

The 1D_201215487 marker was localized to chromosome 1D of *Avena sativa* at position 201,215,487 bp. A potential candidate gene named *1D_gene11508* was detected at 28.2 kb downstream from this marker. According to BLASTP comparison, *1D_gene11508* shared 66.67% similarity with a zinc finger MYM-type 1-like protein of *Triticum dicoccoides*.

The associated marker 1D_461680555 was also localized on chromosome 1D at position 461,680,555 bp. One potential candidate gene was identified 14.3 kb upstream of this marker. This gene, *1D_gene55679*, is likely to encode the ervatamin-B-like gene since it shares 73.96% similarity with the *Lolium rigidum* ervatamin-B-like gene.

The marker 2A_297564389 was localized to the 2A chromosome at position 297,564,389 bp. A potential candidate gene named *2A_gene19938* was detected at 10.7 kb upstream from this marker. This gene showed similarity with the Pimeloyl-ACP methyl ester carboxylesterase of *L. rigidum* which was validated by BLASTP.

The 5C_33721066 marker was localized to chromosome 5C of *A. sativa* at position 33,721,066 bp. A potential candidate gene named *5C_gene3312* was detected at 21.9 kb upstream from this marker. According to BLASTP comparison, *5C_gene3312* shared 88.1% similarity with the ACT domain-containing protein ACR4-like protein of *L. rigidum*.

The marker 4C_270343673 was localized to the 4C chromosome at position 270,343,673 bp. The closest gene named *4C_gene32033* was detected at 9.7 kb downstream of the associated marker. This gene showed similarity with the hypothetical protein EJB05_32782 of *Eragrostis curvula* which was validated by BLASTP.

The candidate genes closest to the three SNP loci associated with flag leaf length within the given interval were *6A_gene22964*, *7A_gene17036*, and *5A_gene1085*, which were annotated as *putative aquaporin PIP2-2*, *triacylglycerol lipase OBL1-like*, and *scarecrow-like protein 21* (**Table 1**) by BLASTP comparison, respectively.

The marker 6A_276363495, associated with FLL, was localized to the 6A chromosome at position 276,363,495 bp. The closest gene named *6A_gene22964* was detected at 8.1 kb upstream of the associated marker. This gene showed similarity with the *putative aquaporin PIP2-2* of *Triticum urartu* which was validated by BLASTP.

The 7A_90499576 marker was localized to chromosome 7A of *A. sativa* at position 90,499,576 bp. A potential candidate gene named *7A_gene17036* was detected at 52.5 kb downstream from this marker. According to BLASTP comparison, *7A_gene17036* shared 81.12% similarity with a triacylglycerol lipase OBL1-like protein of *Lolium perenne*.

The associated marker 5A_9808941 was localized on chromosome 5A at position 9808941 bp. One potential candidate gene was identified 102.7 kb downstream of this marker. This gene, *5A_gene1085*, is likely to encode scarecrow-like protein 21 since it shares 89.76% similarity with *L. rigidum* scarecrow-like protein 21.

### Core collection development

To develop a core collection, 10% and 15% of the lines were sampled from the whole collection, respectively. According to the allele coverage (CV) analysis, CV values of 10% and 15% core collection were 0.9975 and 0.9990, respectively (**Supplementary Figure S3**). We proposed the 15% subset as the core collection since this subset covered more alleles of the oat germplasm, and the size of this core collection is more suitable for use in breeding. The lines of the core collection are listed in **Supplementary Table S4**. PCA distribution of the samples selected in the core collection compared to the whole collection is shown in **Supplementary Figure S4**. The core collection members were evenly distributed throughout the entire collection. This core collection can then be directly used for forage breeding or improved through breeding procedures.

## Discussion

The agronomic traits of forage oats exhibit phenotypic variations that are typically influenced by multiple genes or quantitative trait loci (QTLs), exerting a broad impact on the plant’s growth, development, and ultimately, grass yield. The demand for crop varieties with specific agricultural properties drives the necessity for advancing breeding techniques. Forage oat breeding has historically relied on selecting for morphological, physiological, and chemical traits. However, the modern approach of MAS necessitates an integrated understanding of both genetic and phenotypic data. GWAS represents a robust method for uncovering high-density SNPs, pinpointing genomic regions and genes linked to agronomic traits. This technique is essential for elucidating the complex genetic architecture underlying these traits in diverse germplasm collections, thereby enabling more precise and efficient crop improvement strategies. SLAF-based GBS enables detailed genetic profiling, genome-wide association studies, linkage studies, and genomic mapping using SNPs as markers. Consequently, in our research, we analyzed 340 forage oat genotypes employing an SLAF-based GWAS approach to comprehensively explore the genetic relationships influencing approximately eight key traits.

### Abundant phenotypic diversity and variation in 340 forage oat genotypes

The large amount of variation and phenotypic diversity in these eight descriptive traits observed among panels indicated abundant genetic diversity among the genotypes. Flag leaf is one of the most functional leaves. It is important to genetically analyze the morphological characteristics of functional leaves, especially flag leaf in oat improvement. The CV and H of oat germplasm resources showed a higher variation in FLW (**Supplementary Table S1**). This suggested that genetic diversity is higher in FLW than in other traits. For the same trait, the H values of our germplasm resources were similar in all four environments (**Supplementary Table S1**). This implied that our phenotype data are consistent under the four different environments. In general, the genetic diversity of these agronomic traits in 340 genotypes was shown to be abundant and cover extensive genetic variation. The results indicated that phenotypic variation of the eight traits was mainly controlled by genetic factors and is suitable for trait–marker association studies.

Previous studies have shown that the phenotypic variations in forage oats were greatly influenced by their growth environment (Huang et al., 2020). The results of this study are consistent with previous findings. There were significant variations among different germplasm materials (**Supplementary Table 1**). Further analysis revealed significant phenotypic correlations among seven traits, excluding stem diameter, across different years and locations (*P <* 0.05) with *R* ^2^ ranging from 0.14 to 0.59 (**Figure 1**). In 2022, no significant correlation was observed between stem diameter of materials in Beijing and Pingluo. This may be attributed to the considerable variation in stem diameter between different tillers of experimental plants, leading to substantial measurement errors. Consequently, the measured values failed to reflect the stem diameter of forage oats in this environment accurately, resulting in poor correlation between different locations.

Furthermore, this study employed strip sowing. Compared to the phenotype values obtained from spot seeding, the differences in phenotype between germplasm materials under strip sowing conditions may be reduced, thereby increasing the difficulty of identifying phenotype and genotype-associated loci. However, the phenotype values of plants under strip sowing conditions more accurately reflect their true performance under field conditions, so the identified SNP loci may possess higher practical application value.

### Effective population structure analysis enabled by high-quality SLAF-seq data

The population structure analysis was conducted on 602,572 high-quality SNPs obtained by SLAF-seq technology. The PCA results indicated that the first three principal components explained a total of 14.78% of the genetic variation. The forage oat population structure analyzed in this study was relatively weak, consistent with the findings of Huang et al. (2020) and Zimmer et al. (2020). According to the PCA and Admixture analysis results, most germplasm did not form distinct structural populations. However, the distribution of some germplasm in G1, G2, G3, and G9 on the PCA plot differed significantly from the majority of the germplasm.

With the help of the Admixture software, population structure information was assessed and the CV error was estimated for the number of subpopulations (*K*) ranging from 2 to 10. The results indicated that the minimum CV error occurred when the *K* = 9 (**Figure 4**). The group structure diagram of subgroups changing from 2 to 9 revealed that as the number of subgroups increased, the mixing between groups gradually became more apparent (**Figure 5**). Based on the clustering results, the majority (over 20 samples) of naked oats and a few hulled oat germplasms were grouped together into G9. The SNP information obtained in this study suggests that naked oats and hulled oats were not segregated into two distinct populations. This could be attributed to the possibility that some germplasm resources utilized in this study may have resulted from the hybridization of hulled oats and naked oats.

The germplasm resources tested in this study were collected from several sources. Considering the results of population structure analysis may be associated with the source of lines, we checked out the distribution of lines from the same source in the nine groups (**Supplementary Table S5**). The data showed that the lines from the same source were randomly distributed in the nine groups.

### GWASs reveal putative genes associated with the variation in agronomic traits of forage oat

In traditional crop breeding research, the oat phenotype was heavily influenced by environmental factors and is typically controlled by multiple genes. Due to the scarcity of phenotype markers, molecular marker technology has become a promising tool for breeders.

In this study, based on phenotypic data and SNP information at the whole-genome level of 340 forage oat genotypes, association analysis was carried out to identify the genomic regions significantly associated with the target traits using the BLINK model. The BLINK model is a machine learning approach used in GWASs to analyze large-scale genetic data. BLINK incorporates linear mixed models to account for population structure and relatedness among individuals offering advantages in removing false-positive sites. According to the Q-Q results, the distribution of observed *P*-values was closest to the distribution of expected *P*-values (**Figure 6**), indicating that the results of GWAS analysis performed using the BLINK model in this study were reliable. To reduce the interference of false positives, we choose 8.30^−8^ as the common threshold for this study. Finally, we identified six SNP loci significantly associated with NN and three SNP loci significantly associated with FLL by GWAS analysis. Molecular marker primers could be developed based on the flanking sequences of these SNP markers, which could be used directly for molecular marker-assisted breeding.

Six candidate genes, *hypothetical protein QYE76_059829*, *zinc finger MYM-type protein 1-like isoform X1*, *ervatamin-B-like*, *Pimeloyl-ACP methyl ester carboxylesterase*, *ACT domain-containing protein ACR4-like*, and *hypothetical protein EJB05_32782*, were identified within a 200-kb range upstream and downstream of the SNP loci significantly associated with NN.

The gene associated with the locus 5C_33721066 is the *ACT domain-containing protein ACR4-like*. This gene is located 21.9 kb upstream of 5C_33721066 (**Table 1**). *ACR4* is closely related to the number of tissue cell layers in *Arabidopsis* sepals and ovules (Gifford et al., 2003). In the root system, *ACR4* is involved in controlling the division of cambium cells (De Smet et al., 2008). The function of the *zinc finger MYM-type protein 1-like isoform X1* gene near the locus 1D_201215487 in plants has not been reported. In animal cells, zinc finger MYM-type protein 1 is associated with the metastasis of gastric cancer, and this protein exerts its transcriptional repression function by directly physically binding to the target gene DNA sequence (Yue et al., 2019). The *ZMYM2* gene, which belongs to the same family, is involved in controlling the cell cycle and is essential for G1/S transition in cells (Cibis et al., 2020). Based on the above evidence, it is speculated that oats may regulate stem node number through cell division-related genes such as ACR4-like and MYM-type proteins.

The genes associated with the 1D_461680555 and 2A_297564389 loci are *ervatamin-B-like* and *Pimeloyl-ACP methyl ester carboxylesterase*, respectively. *Ervatamin-B-like* is a member of the papain-like cysteine protease gene (*PLCPs*) found in the latex of the tropical plant *Ervatamia coronaria*. *PLCPs* are classified into clan CA because of the structural similarity of their conserved catalytic residues to papain (Rawlings et al., 2018). *PLCPs* are generated as inactive precursors featuring an autoinhibitory prodomain that prevents unwanted protein degradation (Coulombe et al., 1996). The prodomain can block the access of substrate to the active site and is also involved in protein folding and subcellular targeting (Taylor et al., 1995; Santamarıa et al., 1998). Cysteine proteases are an important class of proteolytic enzymes involved in the programmed cell death (PCD) process of the anther tapetum during the development of many plant organs (Song et al., 2016). Recently, ervatamin-B-like protein is identified as associated with the morphogenesis of walnut (*Juglans regia* L.) stamen development (Li et al., 2024) and photosynthetic gene expression (Alomrani et al., 2021). *UCH1/2*, a *PLCP* gene found in *Arabidopsis*, significantly impacts stem architecture and greatly increases the number of leaves when overexpressed (Yang et al., 2007). *Ervatamin B-like* may play a crucial role in regulating stem node number in oats, since grasses have an equal number of leaves and stem nodes. *PLCPs* also play roles in maintaining the yield of *Arabidopsis* under low nitrogen (LN) conditions (James et al., 2018) and enhancing resistance to salt and drought stress of sweet potato (*Ipomoea batatas* L.) when overexpressing (Chen et al., 2010).

*Pimeloyl-ACP methyl ester carboxylesterase* is a coenzyme involved in carboxylation reactions and is a key gene in the biotin biosynthesis pathway (Lin, 2012). Biotin is an essential vitamin for plants, which can enhance the resistance to carbonate stress (Wang et al., 2020) and modulate primary root growth in *Arabidopsis* (Gibbs et al., 2021). Biotin also can enhance the biomass in algal culture (Varaprasad et al., 2021). The strongest correlation was observed between the 7D_302568573 locus and NN, with numerous transposon genes located approximately 200 kb upstream and downstream of this locus. The only non-transposon gene identified is located 90.1 kb upstream of this locus, which is a putative gene predicted by bioinformatics. Its true existence and function remain unknown.

Leaf is the main photosynthesis organ, and the agronomic traits of leaves are also very important for forage oat due to the close relationships with grass yield. In this research, we identified three SNP loci significantly associated with FLL (**Table 1**). The genes closest to these three SNP loci are *putative aquaporin PIP2-2*, *triacylglycerol lipase OBL1-like*, and *scarecrow-like protein 21*. Scarecrow-like protein 21 (SCL21) is an important transcription factor in plants belonging to the GRAS family. The GRAS family is named after three representative members: GAI (Gibberellin Insensitive), RGA (Repressor of ga1-3), and SCARECROW (Sc) (Pysh et al., 1999). These proteins are closely related to the phytochrome A signaling pathway in *Arabidopsis* and regulate the elongation of the embryonic axis (Torres-Galea et al., 2013). The function of SCL21 in leaves has not been reported, but several studies have revealed the functions of SCL21 on plant morphogenesis. SCL21 acts redundantly with the related PHYTOCHROME A SIGNAL TRANSDUCTION 1 (PAT1) and SCARECROW-LIKE 5 (SCL5) to activate the expression of the *DNA-BINDING ONE FINGER 3.4* (DOF3.4) transcription factor gene, whose expression is in an ETHYLENE RESPONSE FACTOR 115 (ERF115)-dependent manner (Bisht et al., 2023). ERF115 plays a predominant role in the activation of regenerative cell divisions (Heyman et al., 2013). The overexpression of *SCL21* results in a notable decrease in the count of meristematic cortex cells. Co-overexpression of *ERF115* with *SCL21* resulted in a disorganized shoot and a strong inhibition of root growth (Bisht et al., 2023). According to these lines of evidence, it can be assumed that the function of SCL21 on leaf development may be inducible and requires collaboration with other factors to achieve its effects.

*PIP2;2* (*Plasma Membrane Intrinsic Protein 2;2*) may be a potential regulator of FLL traits. It is a subtype of intrinsic proteins located in the plasma membrane, belonging to the PIP2 subfamily. Aquaporin PIP2;2 is associated with traits such as fiber elongation in cotton (Li et al., 2013), root water absorption, drought tolerance in rice (*Oryza sativa* L.), and salt tolerance in barley (*Hordeum vulgare* L.) (Bai et al., 2021; Patel and Mishra, 2021; Sharipova et al., 2022). *PIP2;2* is involved in regulating water flow in plant cells, which is crucial for water management and osmotic pressure regulation in plants (Javot et al., 2003). In maize, the expression level of *PIP2;2* in the elongation zone is higher than that in the mature zone, which strongly indicates that *PIP2;2* is involved in leaf elongation (Maistriaux et al., 2024). Furthermore, overexpression of *PIP2;2* was confirmed to enhance the tolerance of *O. sativa* to mild salt stress, and overexpression of *PIPs* in transgenic tobacco improves plant height and leaf dry weight (Aharon et al., 2003). On the contrary, knockout of either *PIP2;1* or *PIP2;2* results in the leaves of *Physcomitrella patens* being more bent and twisted. In legume species, cell division and expansion are both sensitive to water stress and are both probably involved in the net effect of water stress on leaf size (Gindel, 1968; Bunce, 1977). All these lines of evidence suggest that *OsPIP* genes may play important roles in regulating water homeostasis and leaf size. Therefore, *PIP2;2* may regulate the development of flag leaf by maintaining cell turgor pressure, promoting plant growth, and responding to environmental changes.

Triacylglycerol lipase OBL1 is an enzyme widely present in plants, primarily involved in the metabolism of fatty acids and glycerol. OBL1 participates in lipid-mediated signaling pathways (Testerink and Munnik, 2005). By regulating lipid metabolism and the flux of acyl groups, OBL1 influences various physiological processes such as stress response, growth, and development (Müller and Ischebeck, 2018). For instance, under adverse conditions, OBL1 may help plants adapt to environmental changes by modulating the levels of lipid signaling molecules (Rottet et al., 2015).

In this study, we only identified candidate genes based on their proximity to the nearest SNP markers. Interestingly, we found that there is a *FAR-RED ELONGATED HYPOCOTYL 3* (*FHY3*) gene located 51.3 kb upstream of *OBL1*. *FHY3* was also selected as a candidate gene in a GWAS study of leaf length in *Arabidopsis thaliana* (Zhang et al., 2023). Therefore, there may be some limitations in the criteria used to screen candidate genes. We only determine candidate genes based on their proximity to SNP markers, which may lead to the omission of genes that are functionally related to SNPs but located further away. Furthermore, gene expression and regulation are complex processes involving interactions between multiple genes, transcription factors, and regulatory elements. Therefore, even if a gene is located closest to an SNP marker, it may not necessarily be the key gene influencing the trait. To accurately identify the key genes affecting a trait, it is necessary to conduct a comprehensive analysis incorporating more biological information and experimental evidence.

Apart from flag leaf length and node number, no significant SNPs were identified for the other six phenotypes when applying the Bonferroni correction threshold set at 8.30^−8^. This result may be attributed to several factors, including the large and complex oat genome and the insufficient number of identified SNP loci. After all, SLAF-seq does not yield all SNP locus like genome resequencing technology. On the flip side, environmental variation or trait complexity may also be the reasons because no significant SNP loci were found for other traits. The phenotype of oat plants is influenced by various factors such as genotype, water condition, temperature, and others. For example, oat plants grow taller when there is sufficient rainfall. When the temperature is too high, some genotypes of oats will bloom earlier. Future research will employ advanced sequencing methods to acquire a greater abundance of SNP loci. Subsequent GWAS analyses on existing phenotypes will aim to uncover SNP information associated with additional traits. Additionally, to overcome the limitations of GWAS in the study of complex traits controlled by multiple genes, genomic prediction methods may be a better choice for future breeding researchers. Next, we will continue to analyze the vegetative traits of forage oat by combining GWAS and genome prediction.

SNP markers identified in the study will serve as ideal tools for MAS programs. By being tightly linked to target traits, such as FLL and NN, these markers allow breeders to indirectly select plants with desirable traits through marker detection, rather than relying solely on phenotypic observations. This indirect selection method improves the efficiency of the breeding process and accelerates breeding, as breeders can quickly identify and select plants with the desired genetic characteristics early in the growth cycle of oats.

### Core collection

Due to the vast size and heterogeneity of global germplasm collections, it often becomes challenging to evaluate and utilize them comprehensively. A core collection addresses this issue by providing a manageable sample that represents the genetic variability of a crop species and its relatives. To effectively use and improve our collection through breeding procedures, we assembled a core collection based on genotypic and phenotypic data using Core Hunter 3 software. This 15% core collection not only captured the representation of the total diversity of the entire collection but also had a manageable number of accessions for trait evaluations and oat breeding utilization. Since our germplasm resource population is not large enough, we will continue to collect oat germplasm resources. With the expansion of the germplasm resource bank, the core germplasm population also needs to be continuously updated and expanded. Nevertheless, the construction of this core collection will greatly promote our oat breeding process.

## Conclusion

Here, we associated phenotypes (eight descriptive agronomic traits) and genotypes using 602,572 SLAF-seq-derived SNPs after a precise evaluation of the population structure and genetic diversity of 340 oat germplasms. Through GWASs, several candidate genes linked to nine significant SNPs were identified, and a number of interesting genes were inferred to be functional in the morphological variation of NN and FLL of oats. Future forage oat breeding efforts must make good use of these genomic and genetic resources available for efficient improvement.

## Data Availability

The datasets presented in this study can be found in online repositories. The names of the repository/repositories and accession number(s) can be found in the article/**Supplementary Material**.
