# Supplementary figures and images for: Genome-wide association for multiple quantitative traits in forage oat germplasm based on specific length amplified fragment sequencing
Source: Front Plant Sci. 2025 Feb 20;16:1527635. doi: 10.3389/fpls.2025.1527635 (PMC11882535; doi:10.3389/fpls.2025.1527635)

GLM.NNblup

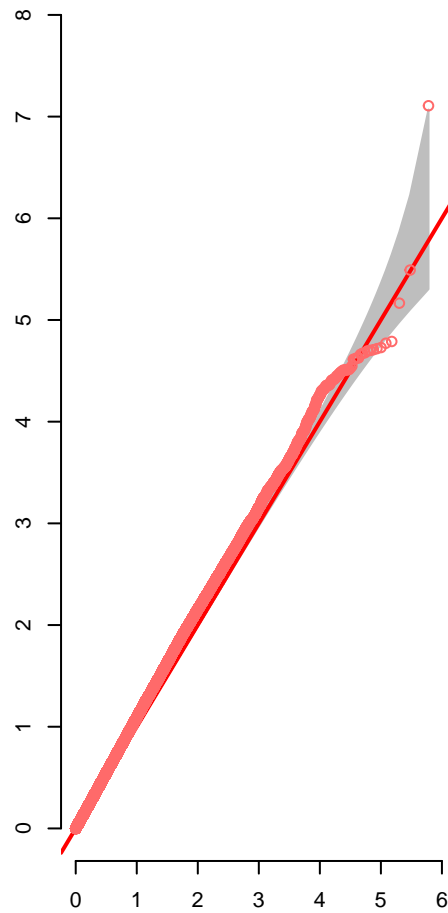

MLM.NNblup

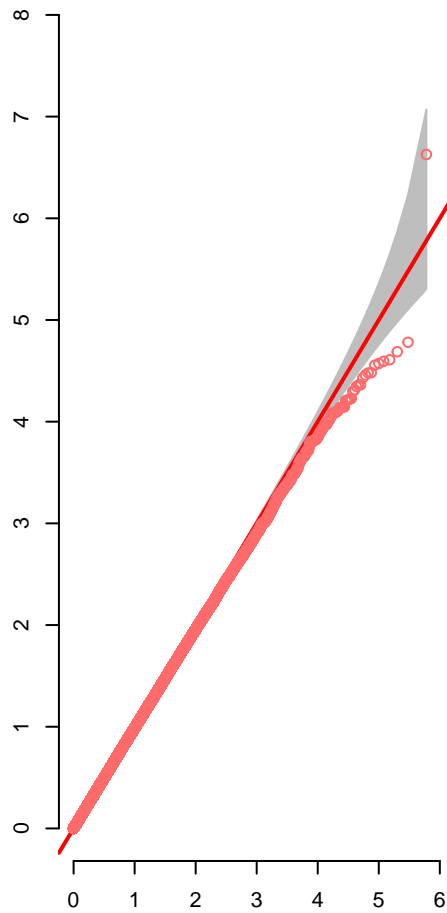

SUPER.NNblup

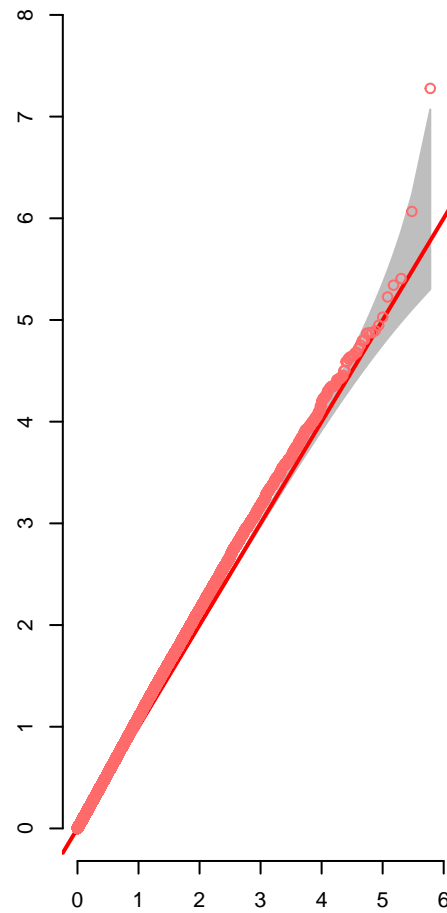

MLMM.NNblup

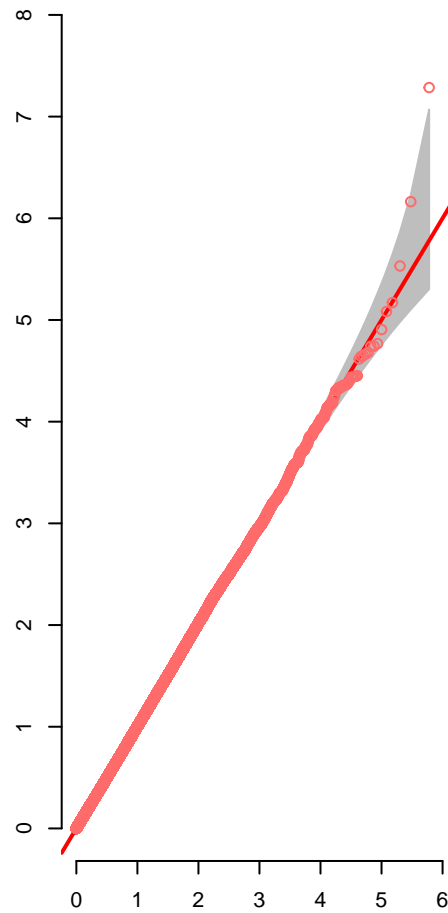

CMLM.NNblup

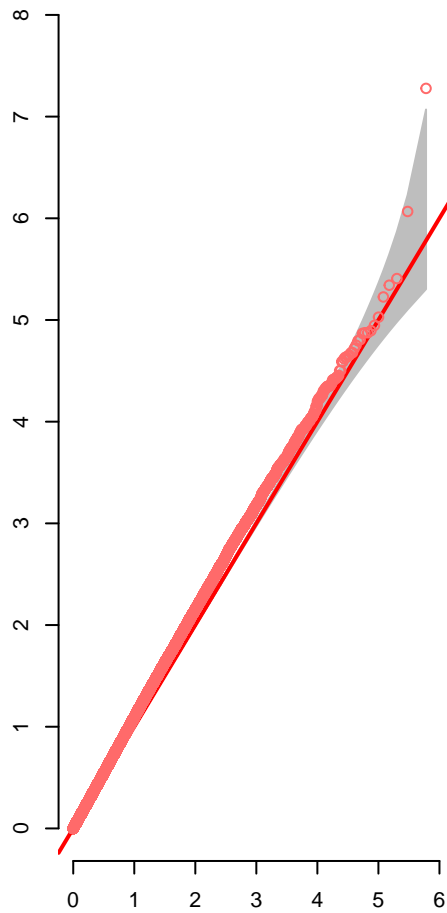

BLINK.NNblup

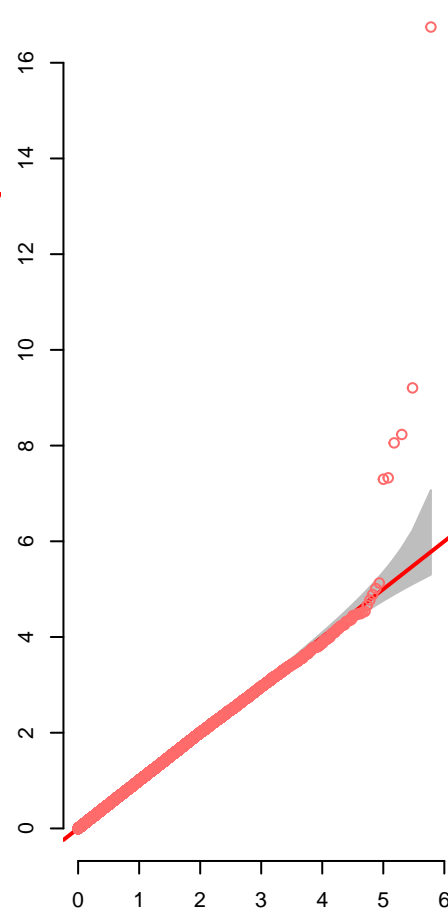

Supplement: Supplementary file 1 [file DataSheet1.pdf]

GLM.FLLblup

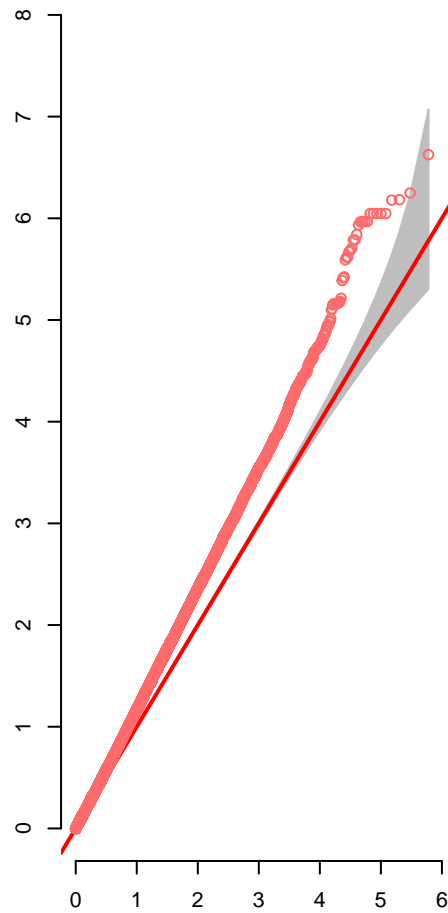

MLM.FLLblup

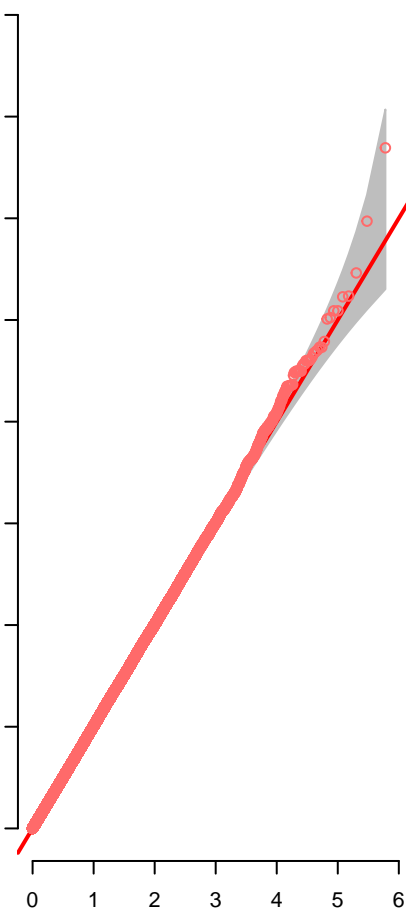

SUPER.FLLblup

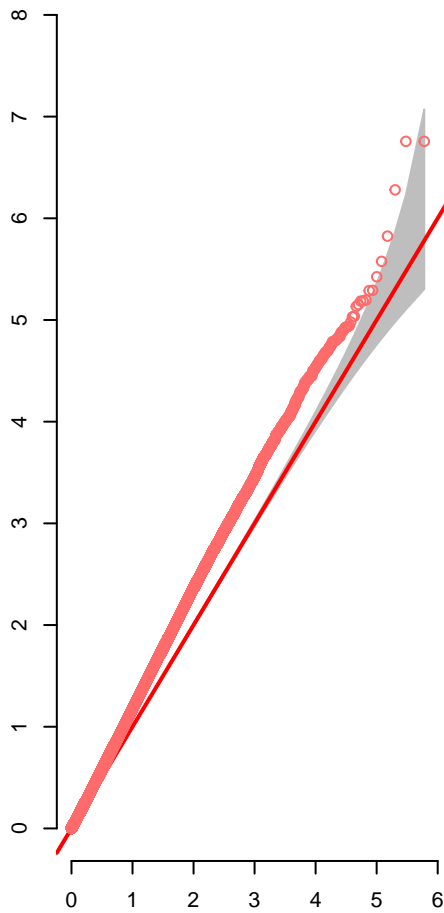

MLMM.FLLblup

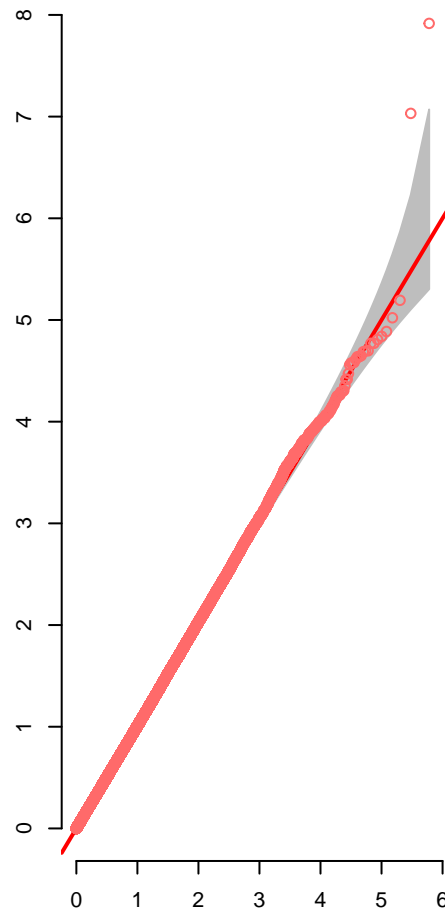

CMLM.FLLblup

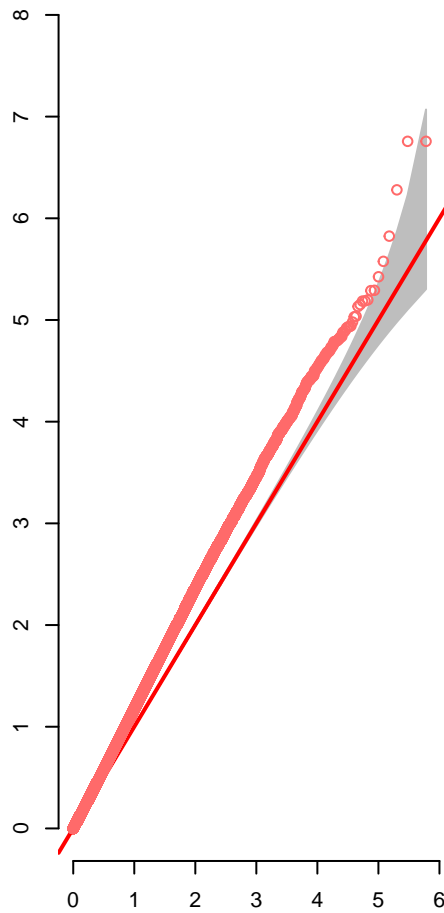

BLINK.FLLblup

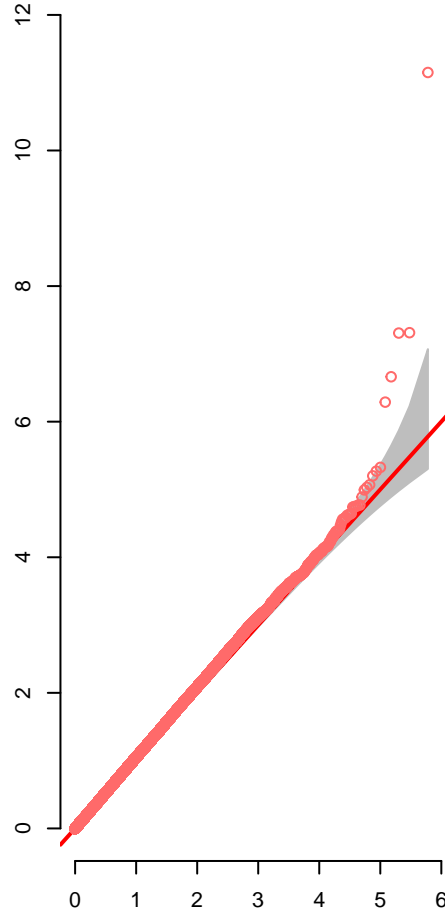

Supplement: Supplementary file 2 [file DataSheet2.pdf]

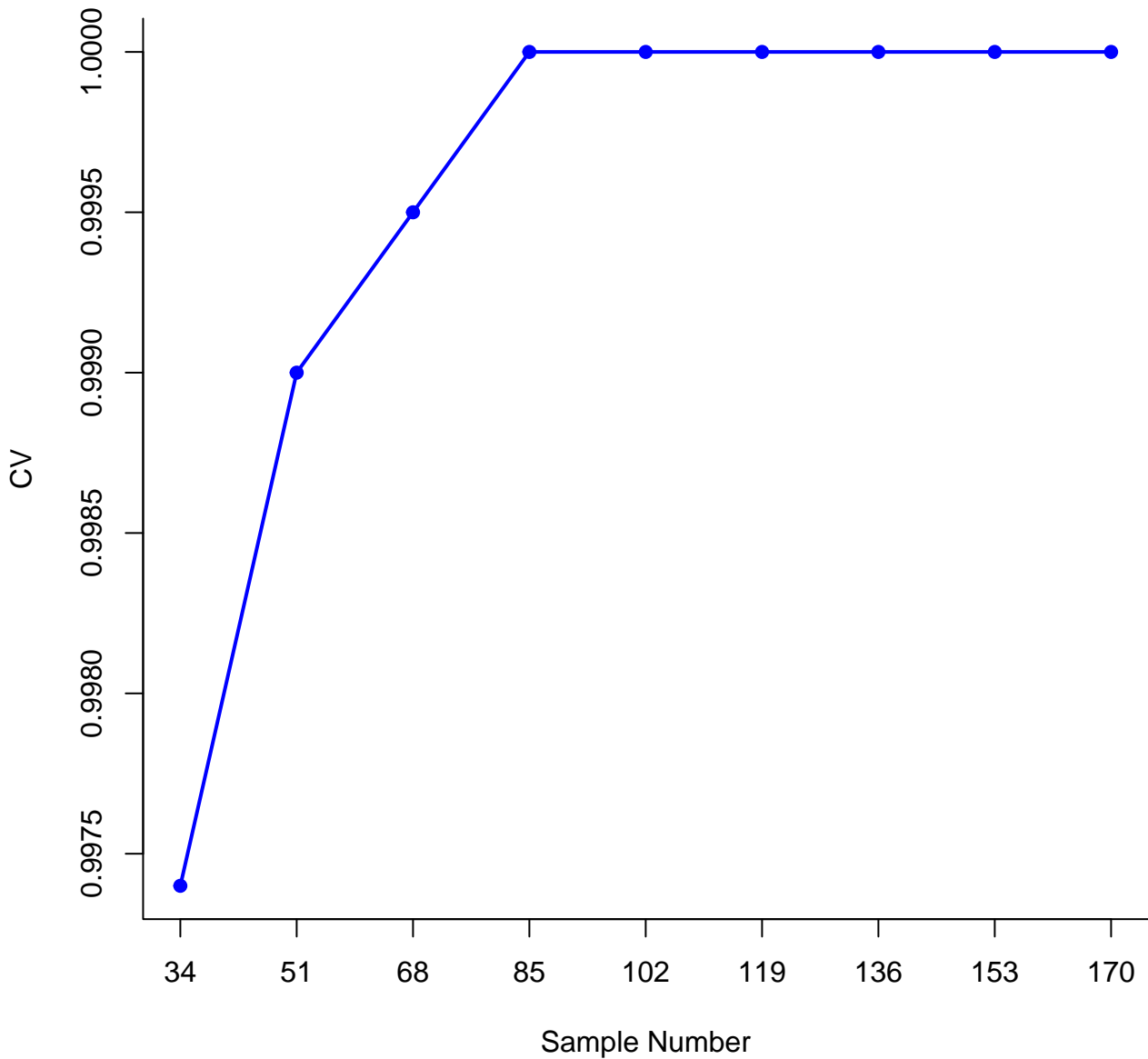

Supplement: Supplementary file 3 [file DataSheet3.pdf]

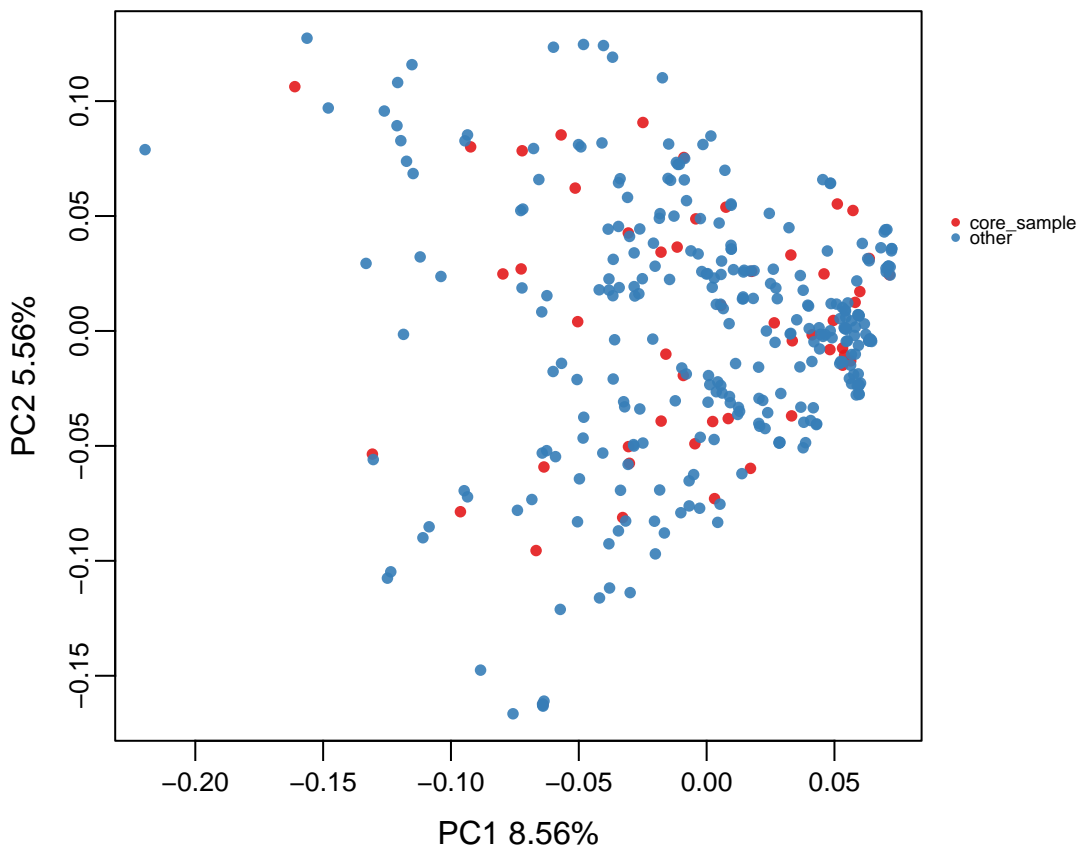

Supplement: Supplementary file 4 [file DataSheet4.pdf]
